# Regulation of Nuclear Receptor Nur77 by miR-124

**DOI:** 10.1371/journal.pone.0148433

**Published:** 2016-02-03

**Authors:** Alexa Tenga, Jordan A. Beard, Apana Takwi, Yue-Ming Wang, Taosheng Chen

**Affiliations:** 1 Department of Chemical Biology and Therapeutics, St. Jude Children’s Research Hospital, Memphis, TN, United States of America; 2 Integrated Biomedical Sciences Program, University of Tennessee Health Science Center, Memphis, TN, United States of America; University of Barcelona, SPAIN

## Abstract

The nuclear receptor Nur77 is commonly upregulated in adult cancers and has oncogenic functions. Nur77 is an immediate-early response gene that acts as a transcription factor to promote proliferation and protect cells from apoptosis. Conversely, Nur77 can translocate to the mitochondria and induce apoptosis upon treatment with various cytotoxic agents. Because Nur77 is upregulated in cancer and may have a role in cancer progression, it is of interest to understand the mechanism controlling its expression. MicroRNAs (miRNAs) are responsible for inhibiting translation of their target genes by binding to the 3ʹUTR and either degrading the mRNA or preventing it from being translated into protein, thereby making these non-coding endogenous RNAs vital regulators of every cellular process. Several miRNAs have been predicted to target Nur77; however, strong evidence showing the regulation of Nur77 by any miRNA is lacking. In this study, we used a luciferase reporter assay containing the 3ʹUTR of Nur77 to screen 296 miRNAs and found that miR-124, which is the most abundant miRNA in the brain and has a role in promoting neuronal differentiation, caused the greatest reduction in luciferase activity. Interestingly, we discovered an inverse relationship in Daoy medulloblastoma cells and undifferentiated granule neuron precursors in which Nur77 is upregulated and miR-124 is downregulated. Exogenous expression to further elevate Nur77 levels in Daoy cells increased proliferation and viability, but knocking down Nur77 via siRNA resulted in the opposite phenotype. Importantly, exogenous expression of miR-124 reduced Nur77 expression, cell viability, proliferation, and tumor spheroid size in 3D culture. In all, we have discovered miR-124 to be downregulated in instances of medulloblastoma in which Nur77 is upregulated, resulting in a proliferative state that abets cancer progression. This study provides evidence for increasing miR-124 expression as a potential therapy for cancers with elevated levels of Nur77.

## Introduction

Nuclear receptors are transcription factors that respond to various stimuli, including growth factors, cytokines, stress, and hormones, and subsequently either promote or repress the expression of their target genes. Nuclear receptors have a variety of important roles within the cell, and deregulation of these receptors can lead to cancer. There are 48 nuclear receptors in humans, many of which are orphans with no known endogenous ligand. Typical nuclear receptors are comprised of an N-terminal DNA-binding domain (DBD) and a C-terminal ligand-binding domain (LBD) that contains a ligand-inducible transactivation function 2 (AF-2) domain [1]. Some nuclear receptors, such as those of the NR4A family, contain another transactivation domain (AF-1) in their N-terminus. These transactivation domains are responsible for recruiting coactivators and corepressors that influence transcription of NR4A target genes either by directly affecting binding of the receptors to DNA or by interacting with other transcription factors [2, 3]. The NR4A family consists of 3 orphan nuclear receptors, Nur77 (NR4A1), Nurr1 (NR4A2), and Nor-1 (NR4A3), which are involved in regulating genes responsible for apoptosis, proliferation, angiogenesis, DNA repair, metabolism, and inflammation [4]. The NR4A genes are characterized as immediate-early response genes that are induced by many signals, including cytokines, stress, and growth factors [5]. The NR4A receptors have roles in cancer development and metastasis, making them ideal targets for the development of anticancer drugs.

Nur77 expression is altered in multiple adult cancers, but little is known about its role in pediatric cancers. Nur77 is expressed in energy-dependent tissues such as skeletal muscle, adipose, and heart [5]. No endogenous ligand has yet been identified for Nur77. It is possible that the activity of Nur77 does not require ligand stimulation since when overexpressed Nur77 is constitutively active even in the absence of stimuli [3]. Many review papers nicely summarize Nur77’s expression in cancer [2, 6, 7], showing that Nur77 is upregulated in many adult cancers, including colon [8–10], bladder [11], pancreatic [12], prostate [13], breast [10, 14], ovarian [10], and lung cancer [10, 15, 16], with the highest expression in melanoma [10]. As summarized in a recent review by Safe et al., Nur77 knockdown in multiple cancer cell lines decreases cell growth and angiogenesis and induces apoptosis, supporting the notion that Nur77 is a pro-oncogenic factor [2]. Furthermore, overexpression of Nur77 in lung cancer cells results in cell-cycle progression and proliferation, which depend on DNA binding and transactivation functions [17].

In contrast to the pro-oncogenic activities just described, the other major function of Nur77 is its translocation to the mitochondria upon induction of apoptotic stimuli, which induces apoptosis by binding to Bcl-2, causing the release of cytochrome c and downstream caspase activation [18]. This mechanism showcases Nur77 as an ideal drug target: several studies have already shown Nur77-mediated apoptosis upon treatment with various compounds, including the natural compound butylidenephthalide (BP), nucleic acid analog 6-mercaptopurine (6-MP) [19, 20], tetradecanoylphorbol-1,3-acetate (TPA), and all-trans retinoic acid (ATRA) [21]. Therefore, Nur77 not only activates the expression of its transcriptional target genes by binding to their promoters in the nucleus, but also induces apoptosis by interacting with Bcl-2 in the mitochondria. Different tissues also display different Nur77 expression patterns. A recent review describes the dichotomous functions, variable expression, and interaction patterns with different cellular signaling pathways of Nur77 [22].

A significant discovery in the last decade is the role of microRNAs (miRNAs) in cancer. These are non–protein-coding endogenous small RNA molecules approximately 22 nucleotides long. They are usually transcribed by RNA polymerase II from either their own genes or from within other introns. After being transcribed into pri-miRNA, they are cleaved by Drosha into pre-miRNA before being exported into the cytoplasm by exportin-5 in a RanGTP-dependent manner. Once in the cytoplasm, they are further processed by Dicer into mature miRNA, which then get incorporated into the RNA-induced silencing complex (RISC). The main function of miRNAs is to regulate gene expression by binding to the 3ʹ untranslated region (3ʹ UTR) of a target mRNA and either destabilizing it or repressing its translation [23, 24]. They can be categorized as both oncogenes (oncomiRs) and tumor suppressors depending upon the genes that they target [25, 26]. A single miRNA can have multiple target genes, and by targeting specific genes, miRNAs play important roles in development, differentiation, proliferation, and apoptosis. Studies over the last several years have shown altered expression of miRNAs in many cancers. The expression level of certain miRNAs can be used as a prognostic biomarker in the diagnosis and monitoring of disease progression [27–29]. The therapeutic potential for miRNAs is being actively explored: in an ongoing clinical trial, a miR-34 mimic, MRX34, is being used to treat liver cancer and several other cancers via liposomal injection [30, 31]. The aberrant expression of miRNAs in cancer may be caused by various mechanisms, including epigenetic modification such as DNA methylation and histone modifications that can modulate miRNA expression [32].

The roles of Nur77 in cancers have been investigated mostly in adult cancers, with very few studies in childhood malignancies. Nur77 is downregulated in leukemia, and Nur77/Nor-1 double-knockout mice quickly develop acute myeloid leukemia before succumbing to the disease [33]; however, the expression and function of Nur77 have not been well studied in pediatric solid tumors. Recent data from the Pediatric Cancer Genome Project show that Nur77 is deleted in many hypodiploid acute lymphoblastic leukemia tumors, whereas it is amplified in some patients with Group 4 medulloblastoma (MB) and rhabdomyosarcoma [34]. To our knowledge, our study is the first to show that the level of Nur77 is regulated by miR-124 and that Nur77 has roles in proliferation of pediatric cancer cells such as Daoy medulloblastoma cells. Most published research on Nur77 as a therapeutic target involves using drugs to induce Nur77-mediated apoptosis; our discovery that miR-124 regulates Nur77 suggests that it may be possible to modulate Nur77 and influence cancer cell growth by regulating miR-124.

## Materials and Methods

### Cell culture

Human embryonic kidney cell line 293T (ATCC CRL-3216), human cortical neuronal cell line HCN-2 (ATCC CRL-10742), human medulloblastoma cell lines D341 (ATCC HTB-187) and Daoy (ATCC HTB-186), and human rhabdomyosarcoma cell lines RD (ATCC CCL-136) and SJCRh30 (ATCC CRL-2061) were obtained from ATCC (Manassas, VA). Cells were free from contamination of mycoplasma, and passaged for fewer than 6 months after receipt (or resuscitation). Human rhabdomyosarcoma cell line Rh41 has been described previously [35]. 293T, HCN-2, and RD cells were grown in Dulbecco’s Modified Eagle’s Medium (DMEM). Rh41 and Rh30 cells were grown in RPMI-1640 Medium, and Daoy cells were cultured in Eagle’s Minimum Essential Medium (EMEM). UKF-NB-3 (NB3) cells, which originated from a patient with *MYCN*-amplified stage 4 neuroblastoma [36], were cultured in Iscove’s Modified Dulbecco’s medium (IMDM). LHCN-M2 cells (immortalized myoblasts derived from the pectoralis major muscle [37]) were plated in gelatin-coated plates (0.1% gelatin in PBS) and cultured in DMEM supplemented with 15% FBS, 0.02M HEPES, 0.03 μg/mL zinc sulfate, 1.4 μg/mL vitamin B12, 0.055 μg/mL dexamethasone, 2.5ng/mL hepatocyte growth factor (recombinant human), and 10 ng/mL basic fibroblast growth factor. All cells were cultured at 37°C in 5% CO_2_, and all media (except LHCN-M2 media) were supplemented with 10% fetal bovine serum, 1% GlutaMAX, 1% sodium pyruvate, and 1% penicillin-streptomycin (Life Technologies, Carlsbad, CA).

### Reporter assay

For the miRNA screen, all miRNA constructs were obtained from an existing library [38]. These constructs are plasmids containing the pre-miRNA sequences and have been reconstituted in TE (10 mM Tris-HCl, pH 8.0; 0.1 mM EDTA, pH 8.0) buffer. Each miRNA construct (0.09 μg) was first added to the well of a 96-well plate (PerkinElmer, Waltham, MA) that were kept at 4°C until all miRNAs were plated. Next, 0.15 μg of Nur77-3ʹUTR reporter plasmid (Nur77-3ʹUTR-Luc; GeneCopoeia, Rockville, MD) and 3 μL/μg of FuGENE 6 (Promega, Madison, WI) were mixed with 50 μL of Opti-MEM reduced-serum media (Life Technologies) and dispensed into each well before being overlaid with 293T cells (20,000 cells/well in 50 μL of antibiotic-free media). Nur77-3ʹUTR-Luc (in the pEZX-MT01 vector) contains both the firefly luciferase gene (*FLuc*) fused upstream of the 3ʹUTR of Nur77 under the control of the SV40 promoter and the *Renilla* luciferase gene (*RLuc*) under the control of the CMV promoter. *RLuc* was used as an internal transfection control. After 48 hours, the Dual-Glo luciferase assay system (Promega) was used to detect luciferase activity according to the manufacturer’s instructions. Raw luciferase activity was measured by using the EnVision 2101 Multilabel Plate Reader (PerkinElmer). Raw values were normalized by dividing the *FLuc* values by the *RLuc* values and then normalized to the value of either pSIF, an empty vector control for miRNA that contains a scrambled sequence in place of the pre-miRNA sequence [38], or oligo control (Cntrl) in the miR-124 inhibitor assay. The FLuc/RLuc values for pSIF or Cntrl were set as 1. Mutations in the 3ʹUTR of Nur77 that disrupt the binding site of the miRNAs were made by Mutagenex (Suwanee, GA).

### RNA isolation and quantitative real-time PCR

Total RNA, including miRNA, was extracted by using the Qiagen miRNeasy kit (Qiagen, Venlo, Netherlands); the Maxwell 16 LEV simplyRNA Tissue Kit was used with the Maxwell 16 Research Instrument (Promega) for RNA extraction only when miRNA extraction was not needed. RNA was converted to cDNA by using the SuperScript VILO cDNA Synthesis Kit (Life Technologies), and 2 μL of 5X Taqman probes (Applied Biosystems) specific to each miRNA were added to enhance miRNA detection. Target gene mRNA expression was detected by using specific Taqman probes (20X) and quantitated via the 7900HT Fast Real-Time PCR System (Applied Biosystems). *GAPDH* (4352934E) was used as an endogenous control for all gene expression analysis, including *NR4A1* (Assay ID Hs00374226_m1), *E2F1* (Assay ID Hs00153451_m1), *CCND2* (Assay ID Hs00153380_m1), *BIRC5* (Assay ID Hs04194392_s1), *TXNDC5* (Assay ID Hs01046709_mH), *CDK4* (Assay ID Hs01565683_g1), and *STAT5A* (Assay ID Hs00234181_m1). Both *RNU6B* (Assay ID 001093) and *RNU48* (Assay ID 001006) were used as endogenous controls for miRNA expression. *RNU48* was used instead of *RNU6B* to analyze endogenous miRNA expression in the cell lines because *RNU48* had less variable Ct values among cell lines. The probes used to detect miRNA levels (Applied Biosystems) were miR-124-3p (Assay ID 001182), miR-15a-5p (Assay ID 000389), and miR-224-5p (Assay ID 002099). *Gapdh* (4352932E) and *Nr4a1* (Assay ID Mm01300401_m1) mouse probes were used to detect Gapdh and Nur77 expression in mice. snoRNA202 (Assay ID 001232) was used as an endogenous control for miR-124 expression in mice as recommended by Applied Biosystems [39]. The fold-change in expression was calculated by using the comparative Ct (ΔΔCt) method, with the values of controls set to 1. All samples were tested in quadruplicate. The Cancer miRNAs Transcriptome PCR Array (SA-Biosciences, MD) was used to identify potential miRNAs that target Nur77. The array was provided by the manufacturer in a 96-well PCR plate, each well containing cDNA sample synthesized from HeLa cells treated with one of 90 cancer-related miRNA mimics. According to the manufacturer’s instruction, we added qPCR MasterMix and Nur77 probe and performed qPCR. We used the data analysis software provided by SABiosciences to analyze qPCR data and determine which miRNAs affect Nur77 expression.

### miR-124 inhibitor assay

Daoy cells were plated at a density of 100,000 cells per well in 6-well BD Falcon plates (Corning, Corning, NY). After 24 hours, the cells were first transfected with various concentrations of the miR-124-3p inhibitor (single-stranded RNA molecule) or the control oligonucleotide (oligo) for 24 hours at 37°C and then transfected with 1 μg of the Nur77-3ʹUTR-Luc reporter plasmid. Transfecting the miR-124-3p inhibitor first allowed it to sufficiently inhibit the miR-124 activity before transfection of the Nur77-3’UTR-Luc reporter plasmid. After 24 hours of incubation, the cells were reseeded at a density of 4,000 cells per well in a 96-well plate, with 6 wells used for each condition. The DualGlo reporter assay was performed 48 hours later. The miR-124-3p inhibitor used was either the mirVana inhibitor from Life Technologies or the Power inhibitor from Exiqon (Woburn, MA), as indicated in the figure legend; each was used with a control oligo from their respective manufacturers. The Lipofectamine RNAiMAX transfection reagent (Life Technologies) was used with both inhibitors.

### Transfections

Cells were transfected with miRNAs by using Fugene6 (Promega) in Opti-MEM in combination with antibiotic-free media (corresponding to the cells being transfected). The plasmid containing pre-miR-124-1 and its backbone vector pEZX-MR03 were purchased from GeneCopoeia.

For the Nur77 and miR-124 co-transfection assay, Daoy cells were seeded at a density of 100,000 cells per well in a 6-well BD Falcon plate. After 24 hours, cells were transfected with 1 μg Nur77 with or without its 3ʹUTR. Cells were transfected with 2.5 μg miR-124 or its control vector (MR03) 24 hours later and collected for Western blot analysis 48 hours after that.

For Nur77-knockdown assays, Daoy cells were seeded at a density of 250,000 cells in T25 flasks. Once the cells were 60%-70% confluent, they were transfected with 20 nM siNur77 by using Dharmacon siGENOME siRNA (GE Healthcare, Lafayette, CO) and 8 μL of RNAiMAX. After 48 hours, cells were reseeded for viability and proliferation assays. The SMARTpool siNur77 (Catalog # M-003426-04) or the individual siNur77 (Catalog # D-003426-23) was used as indicated in the figure legend. Non-targeting siRNA #4 (Catalog # D-001210-04-20) was used as a control for both the pooled and individual siRNAs.

### Molecular cloning

Nur77 cDNA was cloned into the pEXM12-3XFLAG (N-terminal) vector (GeneCopoeia). Forward (5ʹ– ATACTAGTCCACCATGGACTACAAAGACC –3ʹ) and reverse (5ʹ– ATG AAT TCC TAG AAG GGC AGC GTG TC –3ʹ) primers were used to PCR-amplify 3XFLAG-Nur77 cDNA from the pEXM12-3XFLAG-Nur77 vector. The Nur77 PCR product was then cloned into the pCR2.1 TOPO vector by using the TOPO TA Cloning Kit (Life Technologies). The product was then digested by using SpeI and EcoRI restriction enzymes (New England BioLabs, Ipswich, MA) and ligated into a pSIN-EF2-IRES-Blast lentiviral expression vector to generate pSIN-Nur77. The pSIN vector originated from Addgene (Plasmid #16578, [40]) but was modified by inserting additional enzyme sites and the *BlastR* gene (for resistance to blasticidin).

To generate a pSIN-Nur77-3ʹUTR construct, the 3ʹUTR of Nur77 was cloned from the Nur77-3ʹUTR reporter plasmid (GeneCopoeia) and inserted into the 3ʹUTR region downstream of the Nur77 coding sequence in the pSIN-Nur77 vector. Briefly, the 3ʹUTR sequence was amplified by using forward (5ʹ– ATGAATTCCCCCTGCCTGGGAA –3ʹ) and reverse (5ʹ– ATGGATCCTTTTTTTTTTTTTTTTTTTTTTTTTTTTTTTCCAACTACATGT –3ʹ) primers. This 3ʹUTR PCR insert was electrophoresed on a gel, and the band was gel-purified by using the Qiagen gel extraction kit. The purified 3ʹUTR insert was then cloned into the pCR2.1 TOPO vector (Life Technologies). The TOPO and pSIN plasmids were digested by using EcoRI and BamHI (New England BioLabs), and the 3ʹUTR segment was ligated into the pSIN-Nur77 vector. All primers were synthesized by Invitrogen; all PCR amplifications were performed by using the Phusion High-Fidelity PCR Master Mix with HF Buffer (New England BioLabs); and the sequences of all final DNA constructs were confirmed by performing Sanger sequencing.

### Protein isolation and Western blot analysis

Cells were incubated with Pierce RIPA lysis buffer (Thermo Fisher Scientific, Grand Island, NY) on ice for 20 minutes and centrifuged at 17,500 *g* for 20 minutes. The supernatant was collected, and its protein concentration was measured by using a Pierce BCA Protein Assay kit. Absorbance at 540 nM was measured by using the SpectraMax M5 microplate reader (Molecular Devices, Sunnyvale, CA). The protein was mixed with 10X loading buffer and 4X LDS (Life Technologies), incubated at 95°C for 5 minutes, and loaded into a NuPAGE 4–12% Bis-Tris gel (Life Technologies). The separated protein was then transferred to a nitrocellulose membrane by using an iBlot transfer system (Invitrogen). The blot was blocked at room temperature for one hour by using Odyssey blocking buffer (LI-COR Biosciences, Lincoln, NE). Mouse monoclonal anti-Flag M2 (Sigma; catalog # F1804-5MG; used at 1:1500 dilution) and mouse monoclonal anti-β-actin (Sigma; A5441; used at 1:2000 dilution) antibodies were added and incubated overnight at 4°C. After the primary antibodies were removed, the blot was washed three times with TBST for 15 minutes each time before being incubated with the secondary antibody for 1 hour at room temperature. After 1 hour, the blot was washed three times, and proteins were detected by using the Odyssey imaging system (LI-COR Biosciences). ImageJ [41] was used to measure band intensity.

### Lentivirus production and transduction

Two million 293T cells were seeded into each 10-cm dish. Once the cells reached approximately 90% confluence, they were transfected with 12 μg of the expression plasmid, 3 μg of the VSV-G envelope-expressing plasmid pMD2.G (Addgene, Plasmid #12259), and 6 μg of the 2^nd^-generation lentiviral packaging plasmid psPAX2 (Addgene, Plasmid #12260) by using 60 μL FuGENE6 (Promega) in OptiMEM. Media were replaced with fresh media 24 hours after transfection. The lentivirus supernatant was collected 48 hours after media replacement and filtered through a 0.45-μM filter and titrated by using Lenti-X GoStix (Clontech Laboratories, Mountain View, CA). In cases of low titer, Lenti-X Concentrator (Clontech Laboratories) was used to increase the lentiviral titer.

To exogenously express Nur77, Daoy cells were seeded at a density of 250,000 cells in T25 flasks. Once the cells were 60%-70% confluent, they were transduced with Nur77 lentivirus; Polybrene (AmericanBio, Natick, MA) was used at 0.8 μL/mL to aid transduction efficiency. After 48 hours, cells were reseeded for viability and proliferation experiments.

### Viability assay

Cells were reseeded at a density of 1000 cells per well in 96-well plates, with 5 replicates of each condition. Cell viability was determined by using the CellTiter-Glo Luminescent Cell Viability Assay (Promega): cells were incubated with 100 μL of the reagent for 20 minutes on a shaker, covered with a dark lid. Luciferase activity was measured by using the EnVision 2101 Multilabel Reader (PerkinElmer) on the day the cells were seeded (day 0) and daily after that for 3–4 days. The day 1 through day 4 viability measurements were normalized to that measured on day 0. The initial seeding density of 1000 cells per well was chosen so that cells would be close to but less than 100% confluent by the final day.

### Crystal violet staining

Cells were reseeded in 12-well plates at a concentration of 15,000 cells per well, with 4 replicates of each condition. After removing the media and washing with PBS, we fixed the cells in 4% formaldehyde (Sigma-Aldrich, St. Louis, MO) and gently rocked them for 10 minutes at room temperature. The formaldehyde was then removed, and the cells were washed twice with PBS, incubated with 1% crystal violet (Sigma-Aldrich) while gently rocking for 10 minutes at room temperature, and then rinsed with water until the water washed clear, after which 0.1% SDS was added and incubated for 10 minutes while gently rocking. The absorbance of each well was then measured at 590 nm by using a SpectraMax M5 (Molecular Devices). Crystal violet staining was performed on the same day as the initial cell seeding (day 0) and daily thereafter for 3–4 days. Crystal violet absorbance readings measured on days 1–4 were normalized to that measured on day 0.

### IncuCyte proliferation assays

Cells were reseeded in a 24-well plate at a concentration of 10,000 cells per well, with 4 replicates of each condition. Cell proliferation was monitored by using an IncuCyte live-cell imaging system (Essen BioScience, Ann Arbor, MI): 9 images were captured in each well every 12 hours. The percentage of confluent cells was calculated by using IncuCyte software.

### Stable cell lines

Stable cell lines were prepared by plating 500,000 Daoy cells in 10-cm dishes. After 24 hours, cells in antibiotic-free media were transfected with 10 μg of either pEZX-MR03 (vector control) or pEZX-MR03-miR-124 in FuGENE6 diluted in OptiMEM. The media were replaced with normal growth media 24 hours after transfection. The cells were treated with 1 μg/mL puromycin 24 hours after media replacement. Puromycin was added every 3 days for 2 weeks until nontransduced control cells were completely killed by the puromycin, after which the cells were considered to be stable. Expression of miR-124 in the stable cells was confirmed by using a microscope to observe the GFP signal expressed from the vector and by performing qPCR assays to quantify the levels of miR-124.

### 3D-spheroid formation assay

Parental Daoy cells and Daoy cells stably expressing miR-124 or the control vector (MR03) were seeded into a round-bottom 96-well plate at 3 different densities (288, 800, and 2500 cells/well). Media were changed every 3–4 days, and spheroid areas were calculated after 23 days by using the IN Cell Analyzer 6000 (GE). Viability was also measured on day 23 by using the CellTiter-Glo 3D Cell Viability Assay (Promega) according to the manufacturer’s protocol and shown as raw luminescence units (RLU).

### Cerebellar granule neuron analysis

Cerebellar granule neurons (CGNs) were prepared as described [42]. Briefly, cerebella were dissected from the brains of P7 C57BL/6 mice, and pial layers were removed; the tissue was treated with trypsin/DNase and triturated into a single-cell suspension by using fine-bore Pasteur pipettes. The suspension was layered onto a discontinuous Percoll gradient and separated by centrifugation. The small-cell fraction was then isolated. The resulting cultures routinely contained 95% CGNs and 5% glia. The cultures were maintained in Basal Medium Eagle (BME; Life Technologies) supplemented with glutamine and 10% horse serum. All animal experiments were performed in accordance with a protocol approved by St. Jude Children’s Research Hospital’s Institutional Animal Care and Use Committee. The animals were housed at 22−23°C with a 12 h light/dark cycle and free access to food and water at the St. Jude Animal Resources Center certified by the American Association for Accreditation of Laboratory Animal Care. Animals were euthanized by decapitation for the preparation of CGNs.

### Statistical analysis

Results are shown as the mean ± standard error of the mean. The sample values were compared to control values by using a two-tailed unpaired student’s *t*-test. GraphPad Prism 6 software was used to graph results and to calculate the statistical significance.

## Results

### Three miRNAs directly target Nur77

**Fig 1:**
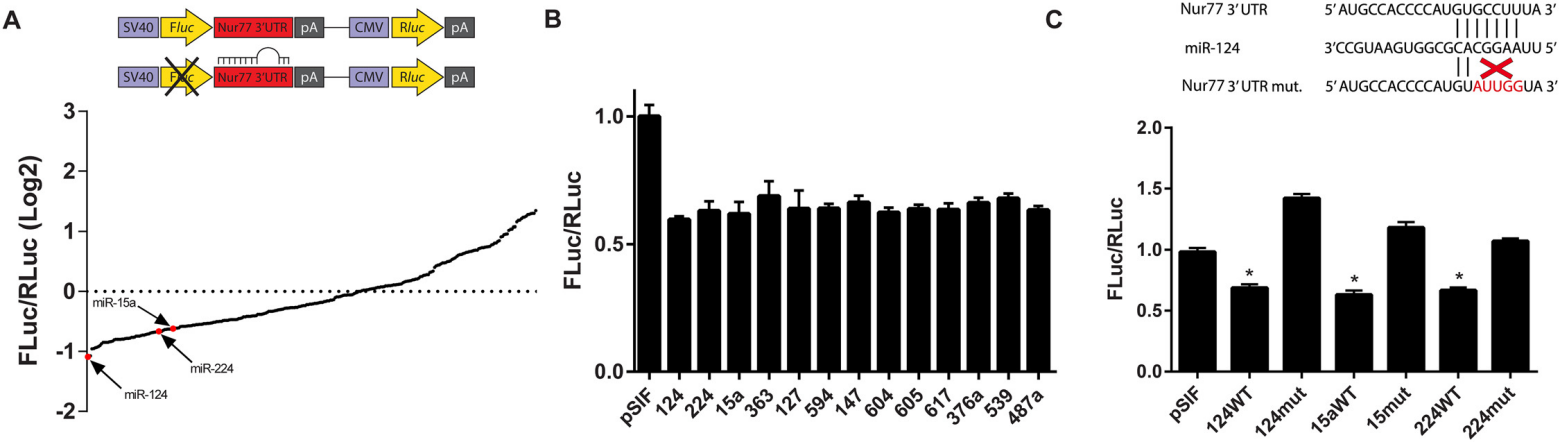
miR-124, miR-15a, and miR-224 directly target Nur77. (A) miR-124 caused the greatest decrease in luciferase activity after 293T cells were transfected with a Nur77-3ʹUTR-Luc reporter and one of 296 miRNAs. Each data point on the waterfall plot corresponds to the resulting luciferase activity for each miRNA. *Renilla* luciferase (RLuc) was used to normalize firefly luciferase (FLuc) activity. (B) Thirteen miRNAs significantly (*p* < 0.001) reduced luciferase activity below that of the pSIF control vector (average of 3 independent experiments is shown). (C) The seed region where miR-124, miR-15a, and miR-224 are predicted to bind within the 3ʹUTR of Nur77 was mutated, and luciferase assays were performed to show direct targeting of Nur77 by these 3 miRNAs. The data shown are the average of 3 independent experiments. * indicates *p* < 0.001.

To identify miRNAs that may target Nur77, we used a luciferase reporter system in which 293T cells were co-transfected with a reporter plasmid containing the 3ʹUTR of Nur77 along with our collection of 296 miRNAs. In the Nur77-3ʹUTR-Luc reporter, the firefly luciferase gene is directly upstream of the 3ʹUTR sequence: a miRNA that binds to the 3ʹUTR will decrease the translation of the luciferase mRNA, resulting in decreased luciferase activity being detected by the Dual-Glo Luciferase Assay System. As shown in a waterfall plot (Fig 1A), miR-124 caused the greatest reduction in luciferase levels among the 296 miRNAs tested. We selected 40 miRNAs that caused 40% or more reduction in luciferase activity and retested them in triplicate: 13 of the 40 miRNAs repeatedly decreased luciferase levels by 30% or more (Fig 1B).

In addition to using the luciferase reporter system, we used the Cancer miRNAs Transcriptome PCR Array (as described in Materials and Methods), which contains cDNA from HeLa cells transfected with one of 90 cancer-related miRNAs, many of which were included in our collection of 296 miRNAs used in the luciferase reporter screen. We found that miR-124 was one of the 3 miRNAs that substantially downregulated Nur77 (S1 Fig).

Among the miRNAs that downregulated Nur77 (Fig 1A and 1B), only miR-124, miR-15a, and miR-224 were predicted by multiple prediction algorithms to target Nur77 by binding to its seed region (a 5- to 8-nucleotide sequence within the 3ʹUTR that mediates the direct binding of a miRNA). These binding predictions are based on the predicted seed regions found within the TargetScan database. When we mutated the binding sites within the 3ʹUTR corresponding to seed regions for miR-124, miR-15a, and miR-224, the mutated 3ʹUTR (124mut, 15mut, and 224mut) became resistant to the corresponding miRNA (Fig 1C), demonstrating that these miRNAs directly target Nur77 by binding to a seed region within the Nur77 3ʹUTR.

### Nur77 is upregulated in pediatric cancer cell lines

**Fig 2:**
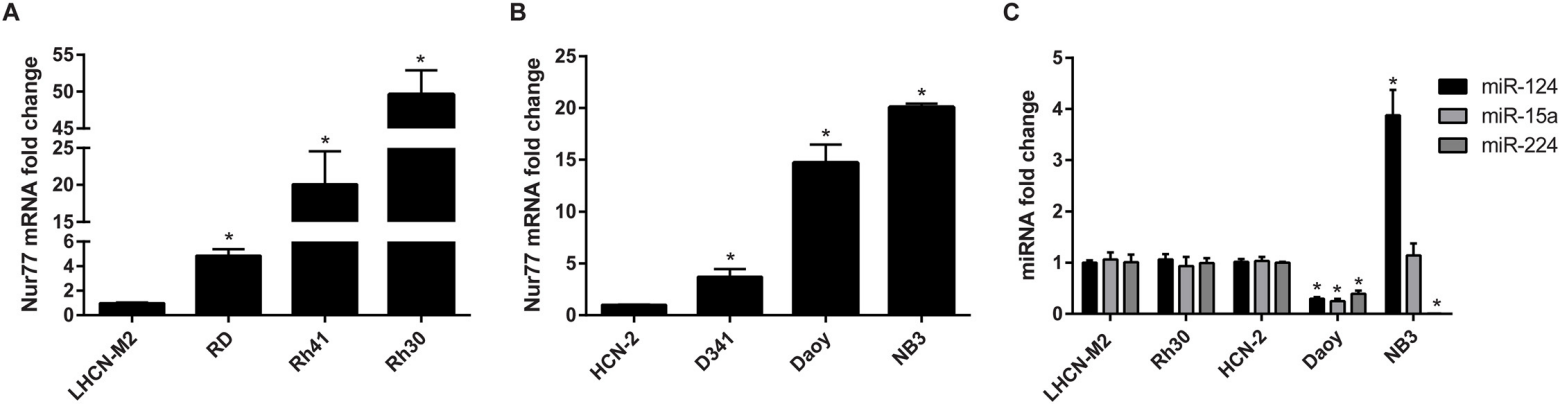
Nur77 is upregulated in pediatric cancer cell lines. (A, B) Nur77 mRNA expression is upregulated in rhabdomyosarcoma, medulloblastoma, and neuroblastoma cell lines. Fold-change was calculated by normalizing the mRNA expression levels to those of their respective control cell lines (either LHCN-M2 or HCN-2), which were set to 1. (C) Endogenous miRNA expression in Rh30, Daoy, and NB3 cells shows that all 3 miRNAs are downregulated in Daoy cells and that miR-224 is downregulated in NB3 cells. RNU48 was used as an internal control. All data shown are the average of 3 independent experiments; *p < 0.0001.

To further investigate the functional relationship between Nur77 and its miRNA regulators, we first analyzed the endogenous Nur77 mRNA levels in several pediatric cancer cell lines. These levels were significantly higher in rhabdomyosarcoma cells lines RD, Rh41, and Rh30 than in LHCN-M2 immortalized myoblasts (Fig 2A). In addition, Nur77 expression in D341 and Daoy medulloblastoma cells and in NB3 neuroblastoma cells was upregulated compared to that of HCN-2 human cortical neurons (Fig 2B). We further analyzed the endogenous expression of miR-124, miR-15a, and miR-224 in Rh30, Daoy, and NB3 cells. As shown in Fig 2C, all 3 miRNAs were downregulated in Daoy, and miR-224 was decreased in NB3 cells. Interestingly, miR-124 was upregulated in NB3, a *MYCN*-amplified neuroblastoma cell line [36], which is consistent with a recent report showing miR-124 upregulation in *MYCN*-amplified neuroblastoma when compared to nine other pediatric solid tumors including rhabdomyosarcoma and non-*MYCN*-amplified neuroblastoma [43]. The mechanism responsible for the upregulation of miR-124 in NB3 cells is unclear. miR-224 was significantly downregulated in NB3 cells (Fig 2C), possibly contributing to the upregulation of Nur77 (Fig 2B). For the remainder of this study, we focused on the relationship between miR-124 and Nur77 in Daoy cells because miR-124 is highly expressed in the brain [44] and it acts as a tumor suppressor in medulloblastoma [45–47]. Furthermore, in undifferentiated granule neuron precursors (GNPs), the level of Nur77 was high but that of miR-124 was low (S2 Fig). Interestingly, once these GNPs differentiated, Nur77 was downregulated and miR-124 was upregulated (S2 Fig). These observations led us to further investigate the functional relationship between miR-124 and Nur77.

### miR-124 decreases Nur77 expression

**Fig 3:**
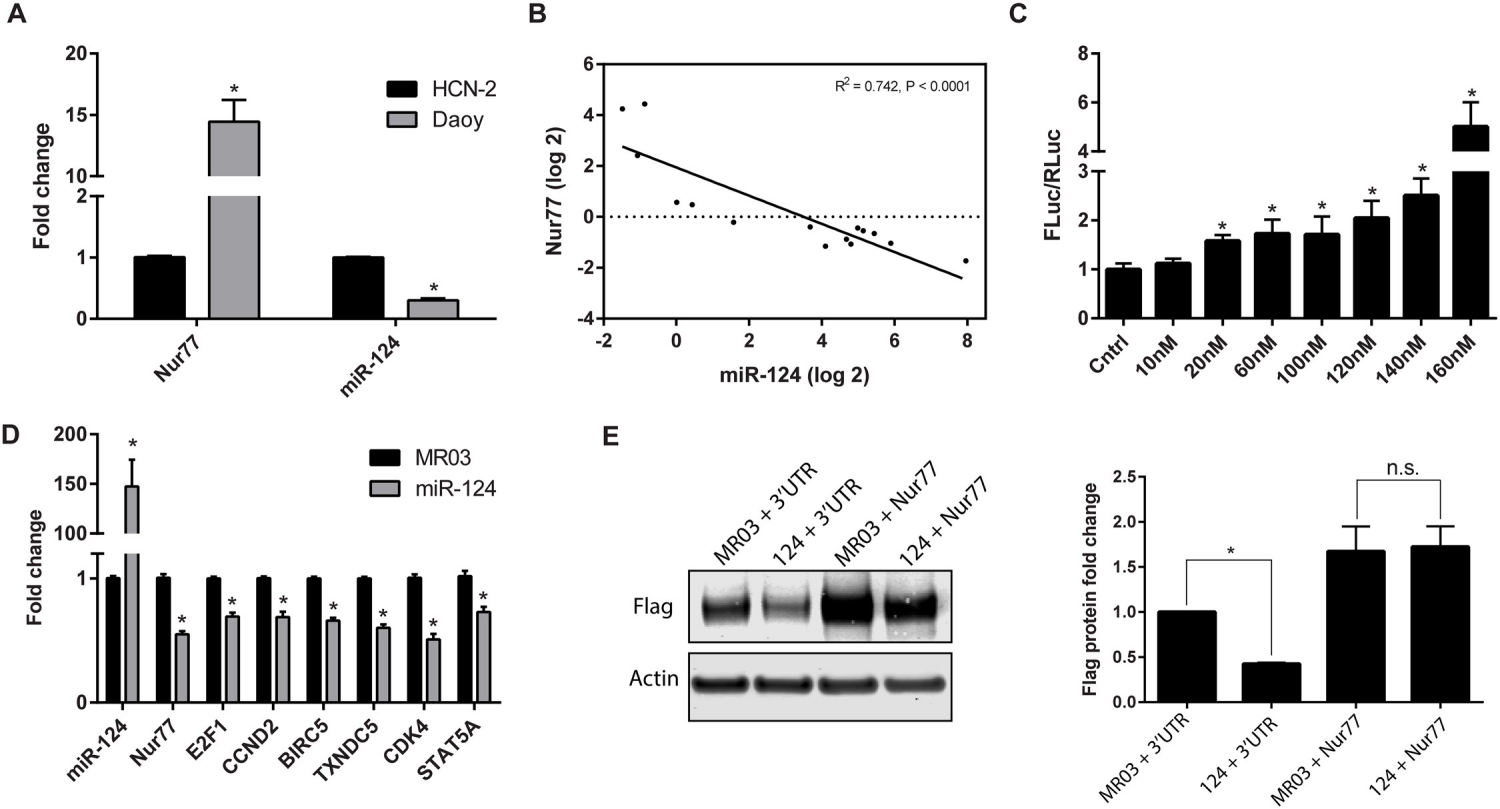
miR-124 decreases Nur77 levels. (A) Endogenous expression levels of miR-124 and Nur77 (mRNA) were measured in Daoy cells and human cortical neurons (HCN-2). (B) Nur77 (mRNA) and miR-124 expression are inversely related in Daoy cells exogenously expressing various levels of miR-124. The levels of Nur77 and miR-124 changed in an inversely correlated manner. (C) Daoy cells were co-transfected with the Nur77-3ʹUTR reporter plasmid (Nur77-3ʹUTR-Luc) and either an inhibitor of miR-124 (10–160 nM of oligonucleotide used as indicated) (mirVana inhibitor from Life Technologies) or an oligo control (Cntrl) (Life Technologies); resulting luciferase levels were measured. The data shown are representative of 3 independent experiments. (D) Either miR-124 or the control vector (MR03) was exogenously expressed in Daoy cells, and the resulting levels of miR-124 and Nur77 (mRNA) were measured along with the expression of Nur77 target genes. (E) Daoy cells were co-transfected with miR-124 (124) or vector control (MR03) and Flag-Nur77 plasmid with (3ʹUTR) or without (Nur77) the 3ʹUTR to confirm that miR-124 targets the 3ʹUTR. Flag and actin protein levels were detected by Western blot and quantified by using ImageJ. Levels of Flag protein were first normalized to those of actin; then MR03-3UTR was set to 1, and all other samples were compared to this sample. The Western blot shown is representative of 3 independent experiments, and the bar graph shows the average protein fold-change from 3 experiments. * indicates *p* < 0.05.

Compared to expression in HCN-2 cells, Nur77 was upregulated and miR-124 was downregulated in Daoy cells (Fig 3A). We further investigated the inverse correlation between Nur77 and miR-124 expression by determining the effect of modulating miR-124 levels on the levels of Nur77. As shown in Fig 3B, higher levels of miR-124 correlated with lower levels of Nur77. Additionally, the Nur77 3ʹUTR-Luc activity increased after treatment with a miR-124 inhibitor, further validating the inverse relationship between miR-124 and Nur77 (Fig 3C). Another miR-124 inhibitor (from Exiqon) was used, yielding similar results (S3 Fig). Exogenous overexpression of miR-124 decreased the mRNA level of endogenous Nur77 and that of several target genes of Nur77, including *E2F1*, *CCND2* (cyclin D2), *BIRC5* (survivin), *TXNDC5*, *CDK4*, and *STAT5A* (Fig 3D). Furthermore, miR-124 overexpression also decreased the expression of Nur77 target genes in 293T cells (S4 Fig). To demonstrate that elevated miR-124 decreases Nur77 protein levels in a 3ʹUTR-dependent manner we examined the effect of overexpressed miR-124 on a Flag-tagged Nur77 construct without the 3ʹUTR (Nur77) or with the 3ʹUTR (3ʹUTR). As shown in Fig 3E and S5 Fig, overexpression of miR-124 decreased the level of Flag-Nur77 only when the 3ʹUTR of Nur77 was present.

### Nur77 promotes cell viability and proliferation

**Fig 4:**
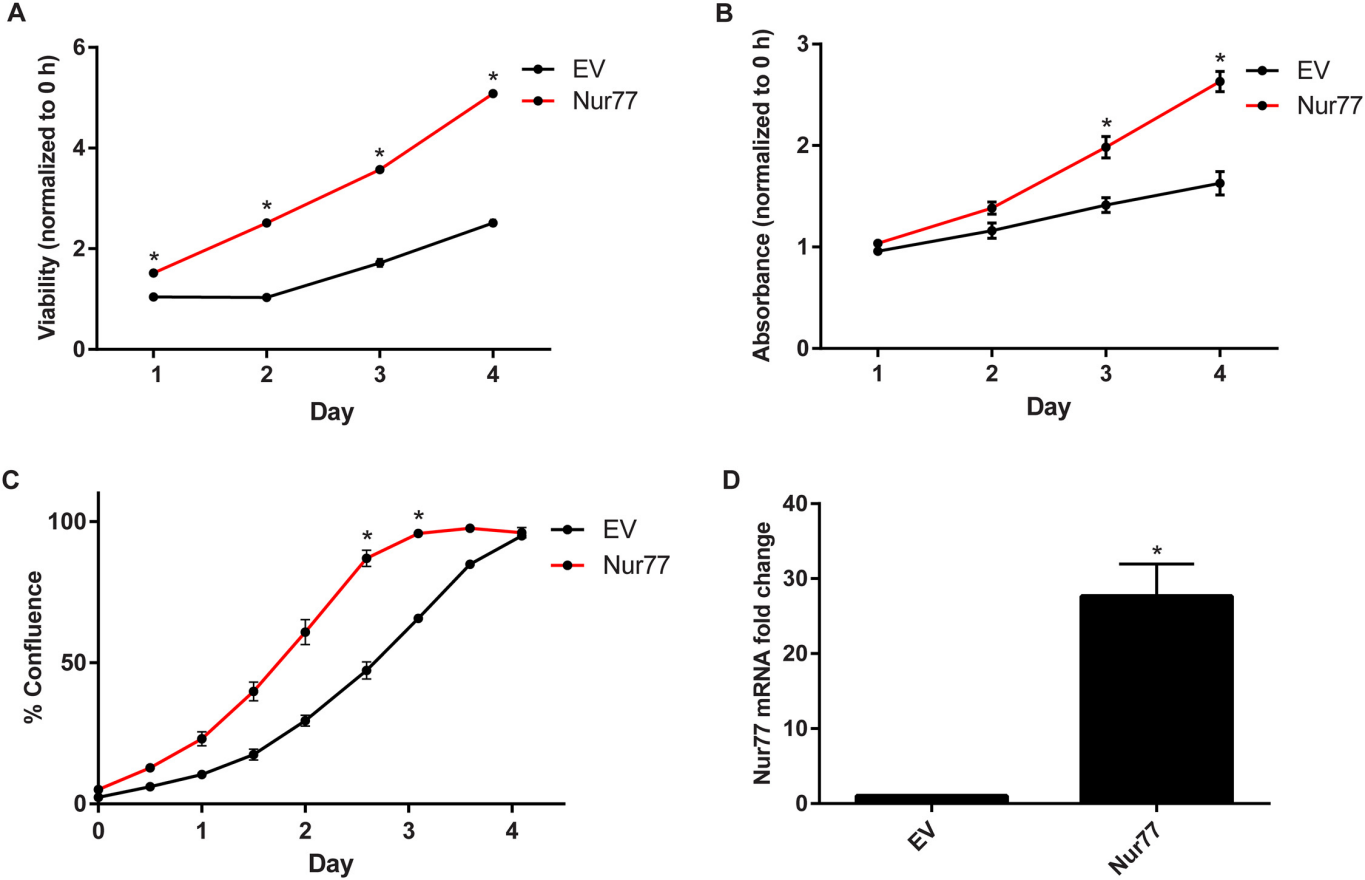
Nur77 promotes cell viability and proliferation. (A) Daoy cells were transduced with pSIN-Nur77 (Nur77) or pSIN vector (EV), and cell viability was measured via the CellTiter-Glo assay every day for 4 days. Viability for each day was normalized to that of Day 0 (0 hours), and statistical significance was calculated for each day. (B) Cells were stained with crystal violet every day for 4 days to measure proliferation over time. The absorbance was measured and normalized to that of Day 0 (0 hours). The statistical significance was calculated for each day. (C) Cell proliferation was monitored by using an IncuCyte live-cell imager for real-time imaging. The resulting cell confluence was recorded every 12 hours for 4 days. (D) Nur77 mRNA level was measured after transduction with Nur77. All experiments were performed by using Daoy cells transduced with EV or Nur77 lentivirus. All data shown are representative of 3 independent experiments; *p≤ 0.0001.

Studies have shown that Nur77 increases cell survival and proliferation in various adult cancer cell lines, suggesting an oncogenic role for Nur77 in those particular cancers [8, 12, 17, 48–50]. We examined whether this was also the case for pediatric cancer cell lines, specifically Daoy medulloblastoma cells. Upon exogenous overexpression of Nur77 in Daoy cells, there was increased cell viability as assessed by the CellTiter-Glo assay (Fig 4A). Cell proliferation was measured by performing crystal violet staining and using an IncuCyte live-cell imaging system, which monitors real-time cell proliferation. Compared to cells transduced with the empty vector (EV), the cells exogenously overexpressing Nur77 showed increased crystal violet staining and confluence over the course of 4 days (Fig 4B and 4C). Fig 4D shows the elevated Nur77 mRNA levels after transduction with Nur77. These data support the notion that Nur77 promotes tumor growth not only in adult cancers but also in pediatric solid tumors.

### Knockdown of Nur77 decreases cell viability and proliferation

**Fig 5:**
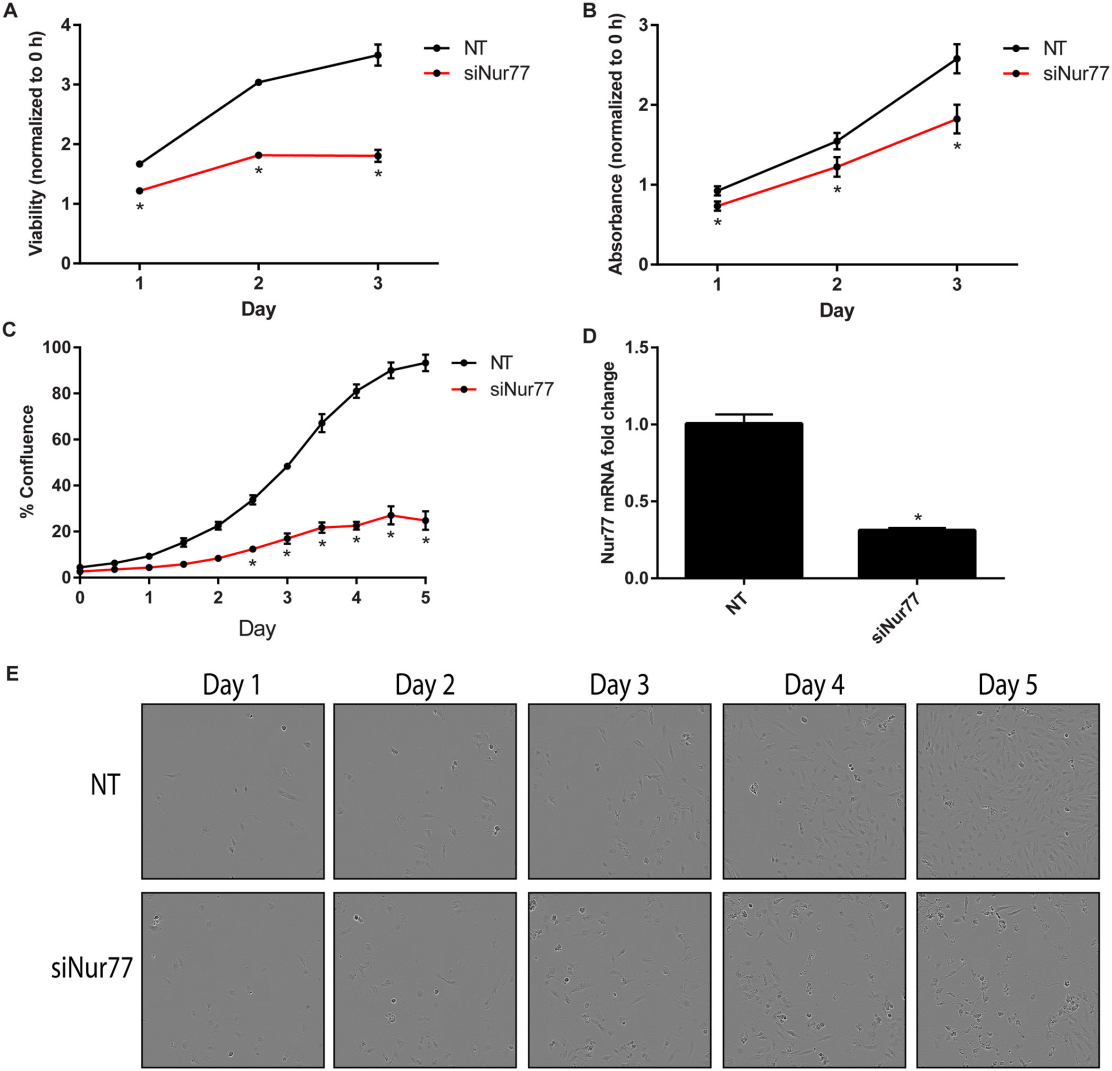
Nur77 knockdown decreases cell viability and proliferation. (A) Daoy cells were transfected with 20 nM siNur77 or non-targeting control (NT), and cell viability was measured via the CellTiter-Glo assay every day for 3 days. Viability for each day was normalized to that of Day 0 (0 hours), and statistical significance was calculated for each day; *p < 0.0001. (B) Cells were stained with crystal violet every day for 3 days to measure proliferation over time. The absorbance was measured and normalized to that of Day 0 (0 hours). The statistical significance was calculated for each day; *p < 0.01. (C) Proliferation was monitored via the IncuCyte live-cell imager. Cell confluence was averaged, with 4 replicates of each condition; *p < 0.0001. (D) Nur77 mRNA was significantly (*p* < 0.0001) decreased after transfecting Daoy cells with siNur77. (E) Images shown for each NT and siNur77 panel over 5 days are the same image view within the same well and are representative of 3 independent experiments with 4 wells for each condition. These images correspond to the data in C. Data shown in A are representative of 5 independent experiments; data in B are representative of 4 independent experiments, and data in C and E are representative of 2 independent experiments. Data shown in D is the average of 4 independent experiments.siNur77, SMARTpool siNur77 (Catalog # M-003426-04) from GE Healthcare.

To further validate the effects of Nur77 on cell proliferation and viability, Nur77 was knocked down via pooled siRNA targeting Nur77 (siNur77). Daoy cells transfected with siNur77 exhibited decreased cell viability and proliferation as measured by the CellTiter-Glo assay, crystal violet staining, and the IncuCyte assay (Fig 5A, 5B, 5C and 5E). Fig 5D shows the knockdown efficiency. The 4 individual siRNAs making up the pooled siRNA were then tested individually. siNur77_4 most efficiently knocked down Nur77 and caused the greatest decrease in cell viability and proliferation (S6 Fig).

### miR-124 decreases cell viability and proliferation

**Fig 6:**
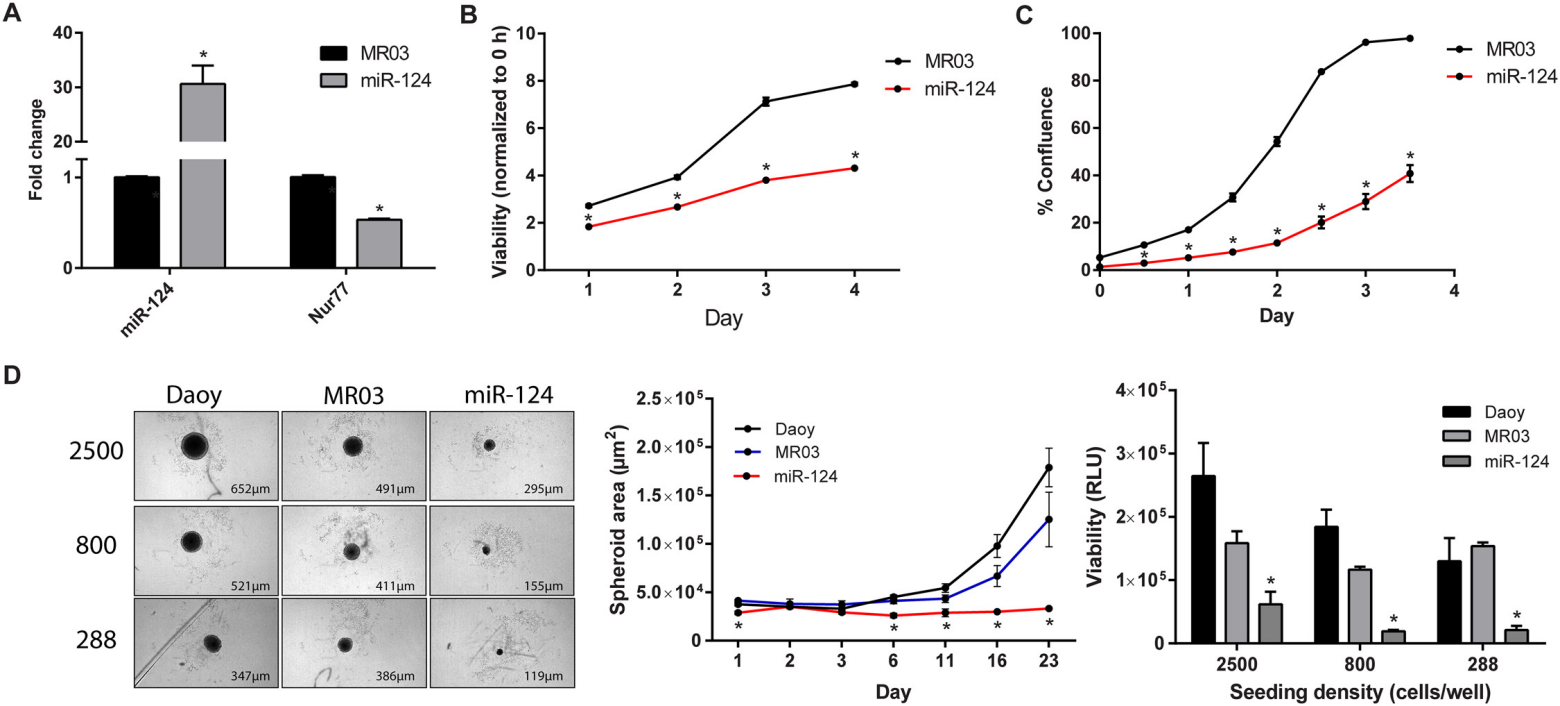
miR-124 decreases cell proliferation in 2D and 3D cultures. (A) Expression of miR-124 was significantly (*p* < 0.0001) increased after antibiotic selection of Daoy cells transduced with pEZX-MR03-miR-124. As a result, Nur77 mRNA levels were significantly decreased (*p* < 0.0001). Data shown are the average of 6 independent experiments. (B) The CellTiter-Glo assay was used to analyze the cell viability of Daoy cells stably expressing exogenous miR-124 or vector control (MR03). Viability for each day was normalized to that of Day 0 (0 hours), and statistical significance was calculated for each day; *p < 0.0001. (C) Stable cells were imaged by using the IncuCyte live-cell imager to determine cell proliferation over the course of 3.5 days, and statistical significance was determined for each day; *p < 0.0001. (D) Parental Daoy cells (Daoy) and Daoy cells stably expressing exogenous miR-124 (miR-124) or its control vector (MR03) were seeded at 3 densities (288, 800, and 2500 cells/well) and grown using 3D culture techniques. After 23 days (left panel), the cells’ spheroid areas (*p < 0.01) were measured by using the IN Cell Analyzer (middle panel). Viability (*p < 0.05) was determined by performing CellTiter-Glo 3D Cell Viability Assays and is shown as raw luminescence units (RLU) (right panel). The spheroid area data shown are for cells seeded at an initial density of 800 cells per well. Data from B are representative of 5 independent experiments; data from C are representative of 4 independent experiments, and data from D are representative of 2 independent experiments.

The observations that Nur77 promoted cell proliferation (Figs 4 and 5) and that miR-124 directly targeted and downregulated Nur77 (Fig 3) led us to examine the effects of miR-124 on cell viability and proliferation. Exogenous and stable expression of miR-124 in Daoy cells substantially reduced cell viability and proliferation (Fig 6A, 6B and 6C). To measure the ability of the Daoy cells to form microtumors, we used round-bottom 96-well plates to promote the formation of 3D spheroids. After allowing the cells to form spheroids for 23 days, we found that cells exogenously overexpressing miR-124 underwent less spheroid growth and viability than did the control cells (Fig 6D). These results are consistent with previously reported evidence showing the negative effects of miR-124 on medulloblastoma cell growth [45–47].

## Discussion

The regulation of Nur77 by miRNAs was previously unknown, and the function of Nur77 in pediatric cancers is currently undetermined. In this study, we found that miR-124 directly targets Nur77 and that Nur77 is upregulated in multiple pediatric cancer cell lines, including rhabdomyosarcoma, neuroblastoma, and medulloblastoma cell lines. We focused on elucidating the function of Nur77 and miR-124 in medulloblastoma cells, and showed that exogenous expression of miR-124 in Daoy medulloblastoma cells decreased the cells’ proliferation and viability.

**Fig 7:**
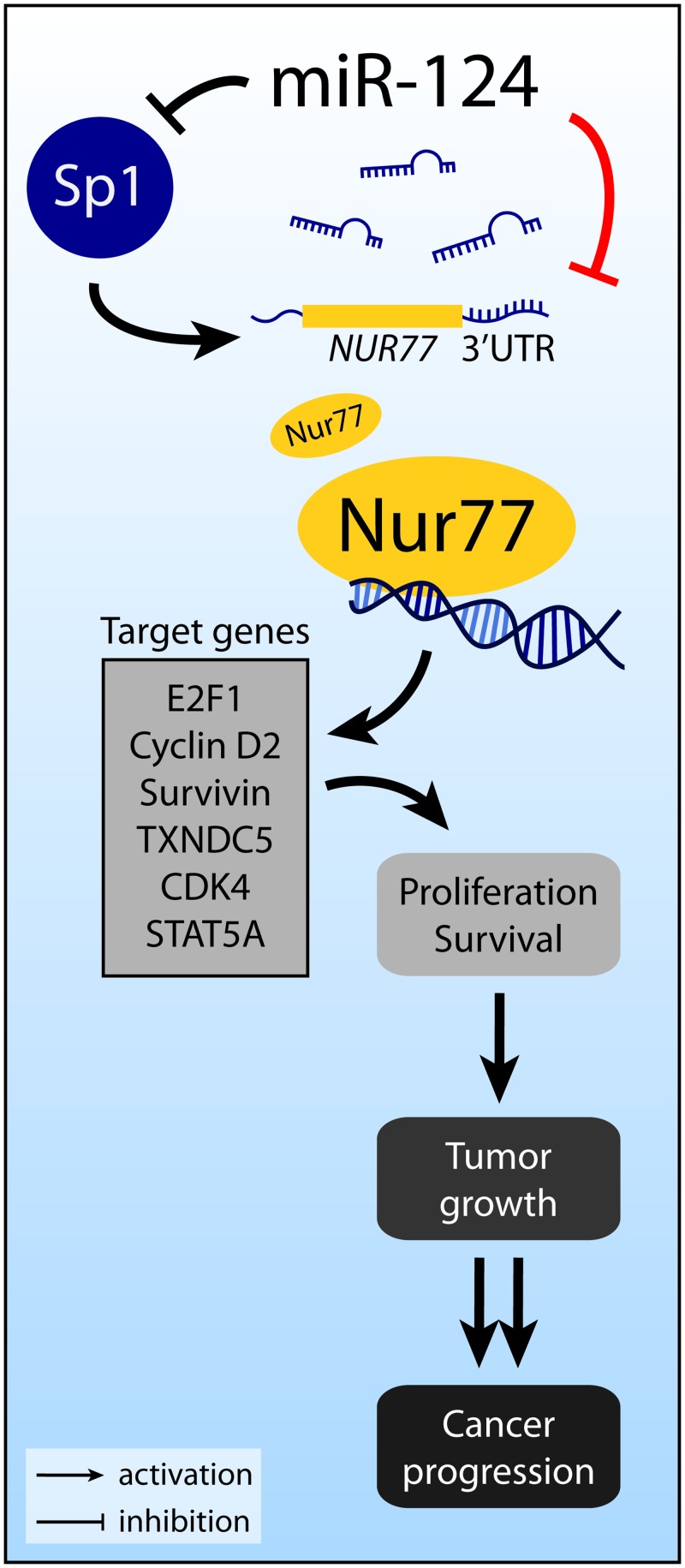
Overview of Nur77 regulation by miR-124. Nur77 can be directly targeted by miR-124, as revealed by our studies reported here (indicated by the red line), or indirectly affected by miR-124 via Sp1. Nur77 may act through several downstream target genes to promote cell proliferation and survival.

Several previous reports suggest that miR-124 might also regulate Nur77 indirectly. In pancreatic beta-cells, Sp1 binds to the promoter of *Nur77* and increases Nur77 levels [51]. Interestingly, during neuronal differentiation of mesenchymal stem cells, miR-124 targets Sp1 mRNA and decreases Sp1 expression [52]. The results of these studies suggest that miR-124 may indirectly decrease Nur77 expression by decreasing Sp1. In addition, Nur77 binds to the promoter of several target genes, including *E2F1*, *CCND2* (cyclin D2), *BIRC5* (survivin), *TXNDC5*, *CDK4*, and *STAT5A* [12, 53–55]. Consistent with these reports, we showed that overexpression of miR-124 decreased expression of these 6 target genes. By promoting expression of these genes, Nur77 exerts its effects on cell proliferation and survival. Aberrant overexpression of Nur77 can, therefore, lead to tumor growth and cancer progression. Fig 7 summarizes our discovery of the miR-124/Nur77 functional relationship in the context of relevant previous reports.

Medulloblastoma is a highly malignant primary brain tumor that originates in the cerebellum. It is also the most common malignant brain tumor in children, with patients having a 50%-80% chance of survival depending on the specific tumor type and other factors [56, 57]. There are 4 subgroups of medulloblastoma: Wnt, Shh, Group 3, and Group 4 [58]. A study profiling miRNAs in Shh MB tumors found 30 miRNAs that were downregulated in tumors with high Gli1, one of which was miR-124 [59]. Another miRNA profiling study also found that miR-124 in a Shh MB mouse model was downregulated compared to that in 1-month-old cerebella [60]. Furthermore, a study profiling 19 human medulloblastomas found that miR-124 was downregulated in the Wnt- and Shh-associated MBs [61]. Additionally, one study of miRNA profiles in 34 human primary medulloblastomas found that miR-124 was downregulated (subtypes were not specified) [62].

Interestingly, miR-124 is the most abundant miRNA in the brain [44] and functions to promote neuronal differentiation [63], regulate neural stem cells [64], and induce differentiation in glioma stem cells [65]. Pierson et al. first showed that miR-124 targets *CDK6* in medulloblastoma cell lines and that miR-124 is downregulated in medulloblastoma cells lines and tumors [45]. Silber et al. went on to show that miR-124 inhibits proliferation of medulloblastoma cells via cell-cycle arrest during G1 and that these results are more dramatic in cells with higher CDK6 levels. Importantly, inducible overexpression of miR-124 *in vivo* significantly reduced tumor growth generated by subcutaneous injection of D425 medulloblastoma cells [47]. Li et al. also found downregulation of miR-124 in 29 medulloblastomas and showed that miR-124 targets *SLC16A1*, which functions to efflux lactic acid during aerobic glycolysis. The authors suggest that inhibition of *SLC16A1* by miR-124 decreases intracellular pH to a lethal level, leading to the observed growth inhibition in medulloblastoma cell lines upon overexpression of miR-124 [46]. It is clear that miR-124 has an important tumor-suppressive role in medulloblastoma and that it acts through various target genes. Our research provides, for the first time, an additional target gene of miR-124, *Nur77*, which has known oncogenic roles in adult solid tumors.

As summarized in Fig 7, miR-124 may also indirectly downregulate Nur77 by directly targeting the mRNA of Sp1, resulting in reduced Sp1 and Nur77 levels. Additionally, miR-124 is predicted to target *CCND2* (which encodes cyclin D2) and *TXNDC5*, which are both target genes of Nur77. Furthermore, miR-124 is predicted to target *RXRA* (RXRα) and *GSK3B* (GSK3β). RXRα and Nur77 heterodimerize and either translocate to the mitochondria to induce apoptosis or bind to the promoters of Nur77 target genes to modulate transcription [18, 66]. GSK3β suppresses Nur77 activity by phosphorylating Nur77 in colorectal cancer [67]. Therefore, it is possible for miR-124 to exert both positive and negative effects directly and indirectly on Nur77, depending on the specific cellular context.

The main type of neuron that makes up the cerebellum is the granule neuron. We found an inverse expression pattern whereby Nur77 is upregulated and miR-124 is downregulated in Daoy medulloblastoma cells and in undifferentiated murine GNPs. Similar to Daoy medulloblastoma cells, in undifferentiated GNPs, the level of Nur77 is high and that of miR-124 is low. Once the GNPs differentiate into mature granule neurons, Nur77 levels drop dramatically and miR-124 expression increases. These observations are consistent with those in studies showing that miR-124 promotes neuronal differentiation [63–65] and that miR-124 levels in 1-month-old mouse cerebellar tissue are higher than those in P6 GNPs [60]. The dramatic decrease in Nur77 expression after differentiation suggests that the levels of Nur77 need to be reduced before the cells can develop into mature neurons.

It is reasonable to hypothesize that increased levels of miR-124 are needed to decrease Nur77 for differentiation to occur. Aberrant downregulation of miR-124 might block differentiation and promote tumorigenesis, warranting the future investigation of the regulation of miR-124 levels. The miR-124 promoter has been reported to be hypermethylated in pancreatic cancer [68], hepatocellular carcinoma [69, 70], ulcerative colitis, [71] and acute lymphoblastic leukemia [72]. It is therefore of interest to analyze the promoter of miR-124 before and after differentiation to identify any changes in methylation status and to identify proteins that may bind to the promoter of miR-124, thereby affecting endogenous levels of miR-124.

Nur77 can reportedly enhance neuronal outgrowth and differentiation: both dibutyryl-cAMP (dbcAMP) and trichostatin A (TSA) promote neurite outgrowth in PC12 rat pheochromocytoma cells by inducing Nur77 expression via acetylated Lys14 of histone H3, and knockdown of Nur77 inhibits dbcAMP and TSA-induced neurite outgrowth [73, 74]. Nur77 overexpression also promotes neurite formation in PC12 cells [74]. However, the opposite phenotype is observed in a murine macrophage cell line. Oxidized low-density lipoprotein (oxLDL) induces mature macrophages to differentiate into dendritic cells and induces Nur77 expression in vascular cells. However, when Nur77 is overexpressed in RAW264.7 murine macrophages, differentiation into dendritic cells is inhibited in the presence of oxLDL [75]. This comes back to the key point that Nur77 functions are heavily dependent on cellular context, so it is possible for Nur77 to have opposing functions in different tissues and cell types.

Daoy cells are classified as desmoplastic cerebellar medulloblastoma [76]; however, researchers have found that this cell line does not mimic any of the 4 subtypes of medulloblastoma. It would be useful to compare the expression level of Nur77 in the brain to that in medulloblastoma tumors, but unfortunately there are not substantial published data showing Nur77 levels in human medulloblastoma. However, two databases show low basal expression of Nur77 in healthy cerebellum. The Brain Transcriptome Database shows that *in situ* hybridization images of the cerebellum have very little Nur77 signal [77]. In addition, the Genotype-Tissue Expression project found that expression levels of Nur77 in different parts of the brain, including the cerebellum, were much lower than those in other normal tissue types [78]. Given our finding of elevated Nur77 in Daoy cells, Nur77 may have an oncogenic role in medulloblastoma, which is supported by our data showing that exogenous overexpression of Nur77 promotes Daoy cell viability and proliferation and that Nur77 knockdown results in the opposite phenotype.

Upon overexpression of miR-124 in Daoy cells, Nur77 mRNA and protein levels and the mRNA levels of Nur77 target genes decreased, showing that miR-124 affects not only Nur77 expression but also the transcriptional activity of Nur77. Stable overexpression of miR-124 led to decreases in cell viability, cell proliferation, and microtumor spheroid size, suggesting therapeutic potential for miR-124 in treating cancer.

## Conclusions

In short, we found that miR-124 targets and decreases Nur77 expression and function. Nur77 promotes cell proliferation in Daoy medulloblastoma cells, but miR-124 reduces it, in part by targeting *Nur77*. This study supports the use of miRNA mimics to treat cancers, especially those in which Nur77 has an oncogenic role.

## Supporting Information

S1 Fig miR-124 decreases Nur77 expression in miRNA array. The Cancer miRNAs Transcriptome PCR Array containing cDNA from HeLa cells transfected with one of the 90 cancer-related miRNAs, as described in Materials and Methods, was used to detect Nur77 expression and identify miRNAs that target Nur77. The resulting gene expression of *Nur77* is displayed as Log2, with horizontal lines indicating the cutoff value (as suggested by the manufacturer) at which *Nur77* gene expression is considered to be significant. Three miRNAs, including miR-124, were found to decrease Nur77 expression.
(DOCX)

S2 Fig Nur77 and miR-124 have inverse expression in granule neurons. Nur77 and miR-124 expression were measured in granule neuron precursors (GNPs) harvested from P7 mice. The GNPs were cultured for 24 hours, allowing enough time for the cells to differentiate (GNP diff.) before being collected for expression analysis. The fold change for the GNPs was set to 1. The internal control for Nur77 was GAPDH, and the control for miR-124 was snoRNA 202. The data shown are the average of 3 independent experiments with the average Ct values indicated below each graph. * indicates *p* < 0.0001.
(DOCX)

S3 Fig An inhibitor of miR-124 increases Nur77 activity. Daoy cells were transfected with the Nur77-3ʹUTR reporter plasmid (Nur77-3ʹUTR-Luc) and either the Exiqon miR-124 Power inhibitor (Exiqon) at the indicated concentrations or the control molecule (Cntrl) (Exiqon), resulting in increased luciferase activity as the concentration of the inhibitor increased. Data shown are representative of 2 independent experiments. * indicates *p* < 0.05.
(DOCX)

S4 Fig miR-124 decreases levels of Nur77 target genes in 293T cells. Transfection of 293T cells with miR-124 decreased the levels of Nur77 and its target genes, *E2F1*, *BIRC5* (survivin), *TXNDC5*, and *CDK4*, compared to those of cells transfected with the vector control (MR03). The data shown are the average of 3 independent experiments. * indicates *p* < 0.01.
(DOCX)

S5 Fig Western blot (uncropped) for Fig 3E. (DOCX)

S6 Fig Nur77 knockdown decreases cell viability and proliferation. (A) Daoy cells were transfected with 20 nM siNur77_4 or non-targeting control (NT), and cell viability was measured via the CellTiter-Glo assay every day for 4 days. Viability for each day was normalized to that of Day 0 (0 hours), and statistical significance was calculated for each day; *p < 0.0001. (B) Cells were stained with crystal violet every day for 4 days to measure proliferation over time. The absorbance was measured and normalized to that of Day 0 (0 hours). The statistical significance was calculated for each day; *p < 0.01. (C) Proliferation was monitored via the IncuCyte live-cell imager. Cell confluence was averaged, with 4 replicates of each condition; *p < 0.0001. (D) Nur77 mRNA was significantly (*p* < 0.0001) decreased after transfecting Daoy cells with siNur77_4. (E) Images shown for each NT and siNur77_4 panel over 5 days are the same image view within the same well and are representative of 3 independent experiments with 4 wells for each condition. These images correspond to the data in C. Data shown in D are the average of 4 independent experiments. Data shown in A and B are representative of 3 independent experiments, and data in C and E are representative of 2 independent experiments.siNur77_4, individual siNur77 (Catalog # D-003426-23) from GE Healthcare.
(DOCX)
