# Supplementary material for: Regulation of Nuclear Receptor Nur77 by miR-124
Source: PLoS One. 2016 Feb 3;11(2):e0148433. doi: 10.1371/journal.pone.0148433 (PMC4739595; doi:10.1371/journal.pone.0148433)
Supplement: S1 Fig — The Cancer miRNAs Transcriptome PCR Array containing cDNA from HeLa cells transfected with one of the 90 cancer-related miRNAs, as described in Materials and Methods, was used to detect Nur77 expression and identify miRNAs that target Nur77. The resulting gene expression of Nur77 is displayed as Log2, with horizontal lines indicating the cutoff value (as suggested by the manufacturer) at which Nur77 gene expression is considered to be significant. Three miRNAs, including miR-124, were found to decrease Nur77 expression. (DOCX) [file pone.0148433.s001.docx]

**Supporting Information**

**
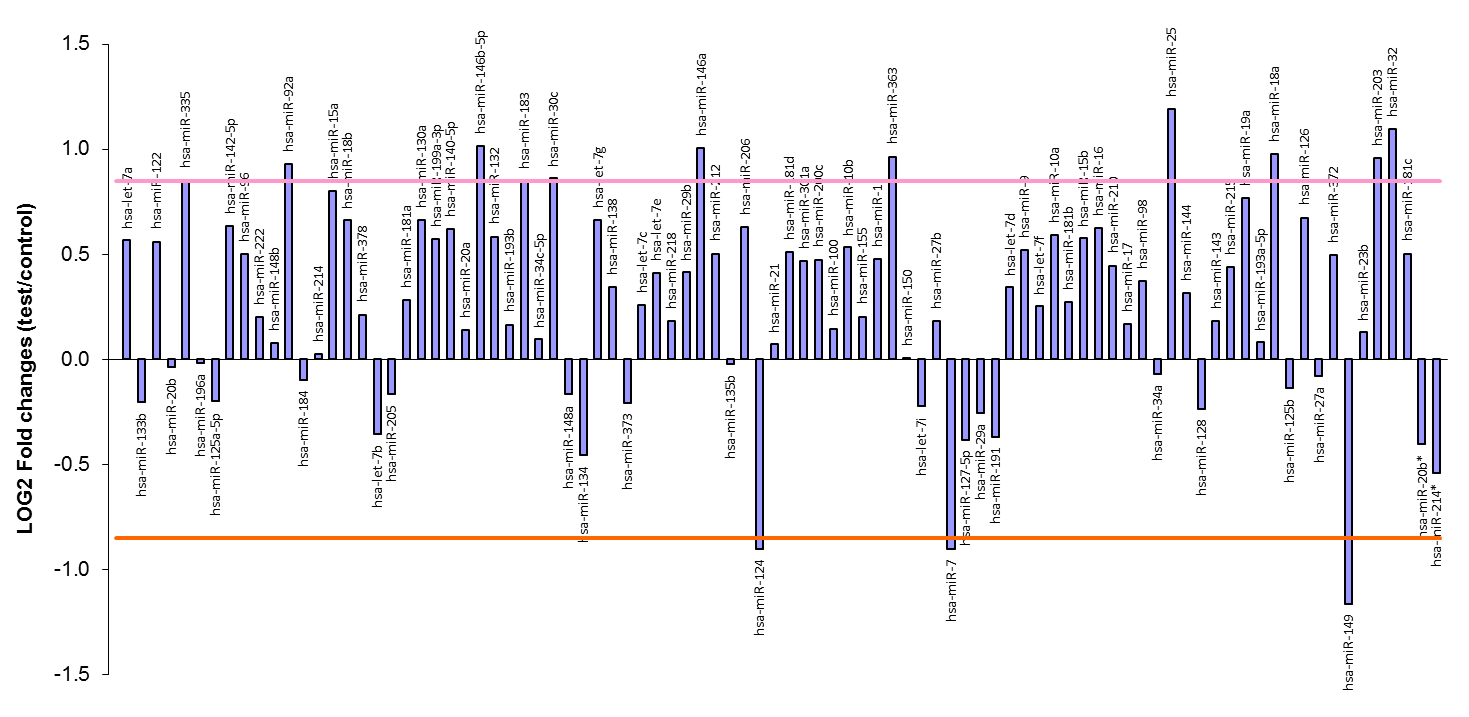
S1 Fig. miR-124 decreases Nur77 expression in miRNA array.** The Cancer miRNAs Transcriptome PCR Array containing cDNA from HeLa cells transfected with one of the 90 cancer-related miRNAs, as described in Materials and Methods, was used to detect Nur77 expression and identify miRNAs that target Nur77. The resulting gene expression of *Nur77* is displayed as Log2 with horizontal lines indicating the cutoff value (as suggested by the manufacturer) at which *Nur77* gene expression is considered to be significant. Three miRNAs, including miR-124, were found to decrease Nur77 expression.
