# Supplementary material for: Regulation of Nuclear Receptor Nur77 by miR-124
Source: PLoS One. 2016 Feb 3;11(2):e0148433. doi: 10.1371/journal.pone.0148433 (PMC4739595; doi:10.1371/journal.pone.0148433)
Supplement: S2 Fig — Nur77 and miR-124 expression were measured in granule neuron precursors (GNPs) harvested from P7 mice. The GNPs were cultured for 24 hours, allowing enough time for the cells to differentiate (GNP diff.) before being collected for expression analysis. The fold change for the GNPs was set to 1. The internal control for Nur77 was GAPDH, and the control for miR-124 was snoRNA 202. The data shown are the average of 3 independent experiments with the average Ct values indicated below each graph. * indicates p < 0.0001. (DOCX) [file pone.0148433.s002.docx]

**Supporting Information**

**S2 Fig. Nur77 and miR-124 have inverse expression in granule neurons.** Nur77 and miR-124 expression were measured in granule neuron precursors (GNPs) harvested from P7 mice. The GNPs were cultured for 24 hours, allowing enough time for the cells to differentiate (GNP diff.) before being collected for expression analysis. The fold change for the GNPs was set to 1. The internal control for Nur77 was GAPDH and the control for miR-124 was snoRNA 202. The data shown are the average of 3 independent experiments with the average Ct values indicated below each graph. * indicates *p* < 0.0001.
