# Supplementary material for: Regulation of Nuclear Receptor Nur77 by miR-124
Source: PLoS One. 2016 Feb 3;11(2):e0148433. doi: 10.1371/journal.pone.0148433 (PMC4739595; doi:10.1371/journal.pone.0148433)
Supplement: S3 Fig — Daoy cells were transfected with the Nur77-3ʹUTR reporter plasmid (Nur77-3ʹUTR-Luc) and either the Exiqon miR-124 Power inhibitor (Exiqon) at the indicated concentrations or the control molecule (Cntrl) (Exiqon), resulting in increased luciferase activity as the concentration of the inhibitor increased. Data shown are representative of 2 independent experiments. * indicates p < 0.05. (DOCX) [file pone.0148433.s003.docx]

**Supporting Information**

**S3 Fig. An inhibitor of miR-124 increases Nur77 activity**. Daoy cells were transfected with the Nur77-3ʹUTR reporter plasmid (Nur77-3ʹUTR-Luc) and either the Exiqon miR-124 Power inhibitor (Exiqon) at the indicated concentrations or the control molecule (Cntrl) (Exiqon), resulting in increased luciferase activity as the concentration of the inhibitor increased. Data shown are representative of 2 independent experiments. * indicates *p* < 0.05.
