# Supplementary material for: Regulation of Nuclear Receptor Nur77 by miR-124
Source: PLoS One. 2016 Feb 3;11(2):e0148433. doi: 10.1371/journal.pone.0148433 (PMC4739595; doi:10.1371/journal.pone.0148433)
Supplement: S4 Fig — Transfection of 293T cells with miR-124 decreased the levels of Nur77 and its target genes, E2F1, BIRC5 (survivin), TXNDC5, and CDK4, compared to those of cells transfected with the vector control (MR03). The data shown are the average of 3 independent experiments. * indicates p < 0.01. (DOCX) [file pone.0148433.s004.docx]

**Supporting Information**

**S4 Fig. miR-124 decreases levels of Nur77 target genes in 293T cells.** Transfection of 293T cells with miR-124 decreased the levels of Nur77 and its target genes, *E2F1*, *BIRC5* (survivin), *TXNDC5*, and *CDK4,* compared to those of cells transfected with the vector control (MR03). The data shown are the average of 3 independent experiments. * indicates *p* < 0.01.
