# Supplementary material for: Regulation of Nuclear Receptor Nur77 by miR-124
Source: PLoS One. 2016 Feb 3;11(2):e0148433. doi: 10.1371/journal.pone.0148433 (PMC4739595; doi:10.1371/journal.pone.0148433)
Supplement: S5 Fig — (DOCX) [file pone.0148433.s005.docx]

**Supporting Information**

MW MR03 124 MR03 124 MR03 124

Ladder EV EV 3’UTR 3’UTR Nur77 Nur77


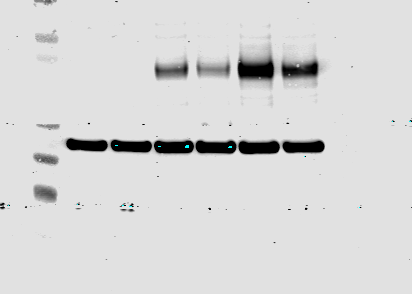


25kD

37kD

50kD

75kD

100kD

Flag

Actin

**S5 Fig.** **Western blot (uncropped) for Fig 3E.**
