# Supplementary material for: Regulation of Nuclear Receptor Nur77 by miR-124
Source: PLoS One. 2016 Feb 3;11(2):e0148433. doi: 10.1371/journal.pone.0148433 (PMC4739595; doi:10.1371/journal.pone.0148433)
Supplement: S6 Fig — (A) Daoy cells were transfected with 20 nM siNur77_4 or non-targeting control (NT), and cell viability was measured via the CellTiter-Glo assay every day for 4 days. Viability for each day was normalized to that of Day 0 (0 hours), and statistical significance was calculated for each day; *p < 0.0001. (B) Cells were stained with crystal violet every day for 4 days to measure proliferation over time. The absorbance was measured and normalized to that of Day 0 (0 hours). The statistical significance was calculated for each day; *p < 0.01. (C) Proliferation was monitored via the IncuCyte live-cell imager. Cell confluence was averaged, with 4 replicates of each condition; *p < 0.0001. (D) Nur77 mRNA was significantly (p < 0.0001) decreased after transfecting Daoy cells with siNur77_4. (E) Images shown for each NT and siNur77_4 panel over 5 days are the same image view within the same well and are representative of 3 independent experiments with 4 wells for each condition. These images correspond to the data in C. Data shown in D are the average of 4 independent experiments. Data shown in A and B are representative of 3 independent experiments, and data in C and E are representative of 2 independent experiments.siNur77_4, individual siNur77 (Catalog # D-003426-23) from GE Healthcare. (DOCX) [file pone.0148433.s006.docx]

**Supporting Information**

**S6 Fig.** **Nur77 knockdown decreases cell viability and proliferation. (A)** Daoy cells were transfected with 20 nM siNur77_4 or non-targeting control (NT), and cell viability was measured via the CellTiter-Glo assay every day for 4 days. Viability for each day was normalized to that of Day 0 (0 hours), and statistical significance was calculated for each day; **p* < 0.0001. **(B)** Cells were stained with crystal violet every day for 4 days to measure proliferation over time. The absorbance was measured and normalized to that of Day 0 (0 hours). The statistical significance was calculated for each day; **p* < 0.01. **(C)** Proliferation was monitored via the IncuCyte live-cell imager. Cell confluence was averaged, with 4 replicates of each condition; **p* < 0.0001. **(D)** Nur77 mRNA was significantly (*p* < 0.0001) decreased after transfecting Daoy cells with siNur77_4. **(E)** Images shown for each NT and siNur77_4 panel over 5 days are the same image view within the same well and are representative of 3 independent experiments with 4 wells for each condition. These images correspond to the data in C. Data shown in D are the average of 4 independent experiments. Data shown in A and B are representative of 3 independent experiments, and data in C and E are representative of 2 independent experiments. siNur77_4, individual siNur77 (Catalog # D-003426-23) from GE Healthcare.
